# Juvenile systemic lupus erythematosus complicated with posterior reversible encephalopathy syndrome: a case report and literature review

**DOI:** 10.1186/s13023-025-04044-0

**Published:** 2025-11-07

**Authors:** Man Luo, Huan He, Qing Zhou, Long Chen, Changming Xia, GuoHua Yuan, Fang He

**Affiliations:** 1 Department of Rheumatology and Immunology, Suining Central Hospital, Suining, 629000 Sichuan China; 2 Department of Radiology and Image, Suining Central Hospital, Suining, 629000 Sichuan China; 3 Department of Pediatrics, Suining Central Hospital, Suining, 629000 Sichuan China; 4 Department of Rheumatology and Immunology, Affiliated Hospital of North SiChuan Medical College, Nanchong, 637000 Sichuan China

**Keywords:** Juvenile systemic lupus erythematosus, Posterior reversible encephalopathy syndrome, Magnetic resonance imaging

## Abstract

**Background:**

Posterior reversible encephalopathy syndrome (PRES) is an acute or subacute neurological syndrome with clinical manifestations including headache, vision loss, altered mental status, and seizures. Magnetic resonance imaging(MRI) can assist in clarifying the diagnosis. Systemic lupus erythematosus (SLE) is an autoimmune disease, and the occurrence of PRES is infrequent, especially in children with SLE.

**Case description:**

Our patient was initially diagnosed with juvenile systemic lupus erythematosus involving the skin, joints, and kidneys. The patient was treated with glucocorticoids and cyclophosphamide, which led to a partial improvement in symptoms. However, the patient experienced neurological symptoms and elevated blood pressure, and head MRI revealed swelling and diffuse damage to the bilateral occipital parietal gyrus. The patient was diagnosed with PRES.

**Results:**

The symptoms were relieved after actively lowering blood pressure, dehydrating with mannitol, and treating with glucocorticoids, cyclophosphamide, and telitacicept. Six months later, no abnormalities were observed in the follow-up head MR image. We searched PubMed to review the characteristics of 15 children with SLE who developed PRES, and most of them recovered after active treatment.

**Conclusion:**

Various factors can lead to complications of PRES in paediatric patients with systemic lupus erythematosus. In our patient, the likely cause was the high disease activity of SLE. This review revealed that juvenile SLE cases accompanied by PRES are often associated with lupus nephritis, hypertension, and high disease activity. Most patients recover with timely and active treatment, with only a few experiencing recurrence.

## Introduction

Posterior reversible encephalopathy syndrome (PRES) is an acute or subacute encephalopathy syndrome that primarily manifests as headaches, seizures, and visual disturbances. The main causes of PRES include hypertension, end-stage renal disease, preeclampsia or eclampsia, systemic lupus erythematosus, medications, and the use of immunosuppressants following organ transplantation. MRI is a key tool for its diagnosis. PRES is more common in young adults, with a higher incidence in females than in males, and is even rarer in children [1]. We describe a case of childhood systemic lupus erythematosus (SLE) with active disease complicated by PRES, which improved after treatment. Such cases are relatively uncommon. To further understand the clinical characteristics of PRES in childhood SLE patients, we reviewed 15 reported cases. The main triggering factors were lupus nephritis, renal insufficiency, high disease activity, and hypertension. The secondary triggers included corticosteroid, plasmapheresis, and the use of medications such as cyclosporine.

## Case report

**Fig. 1:**
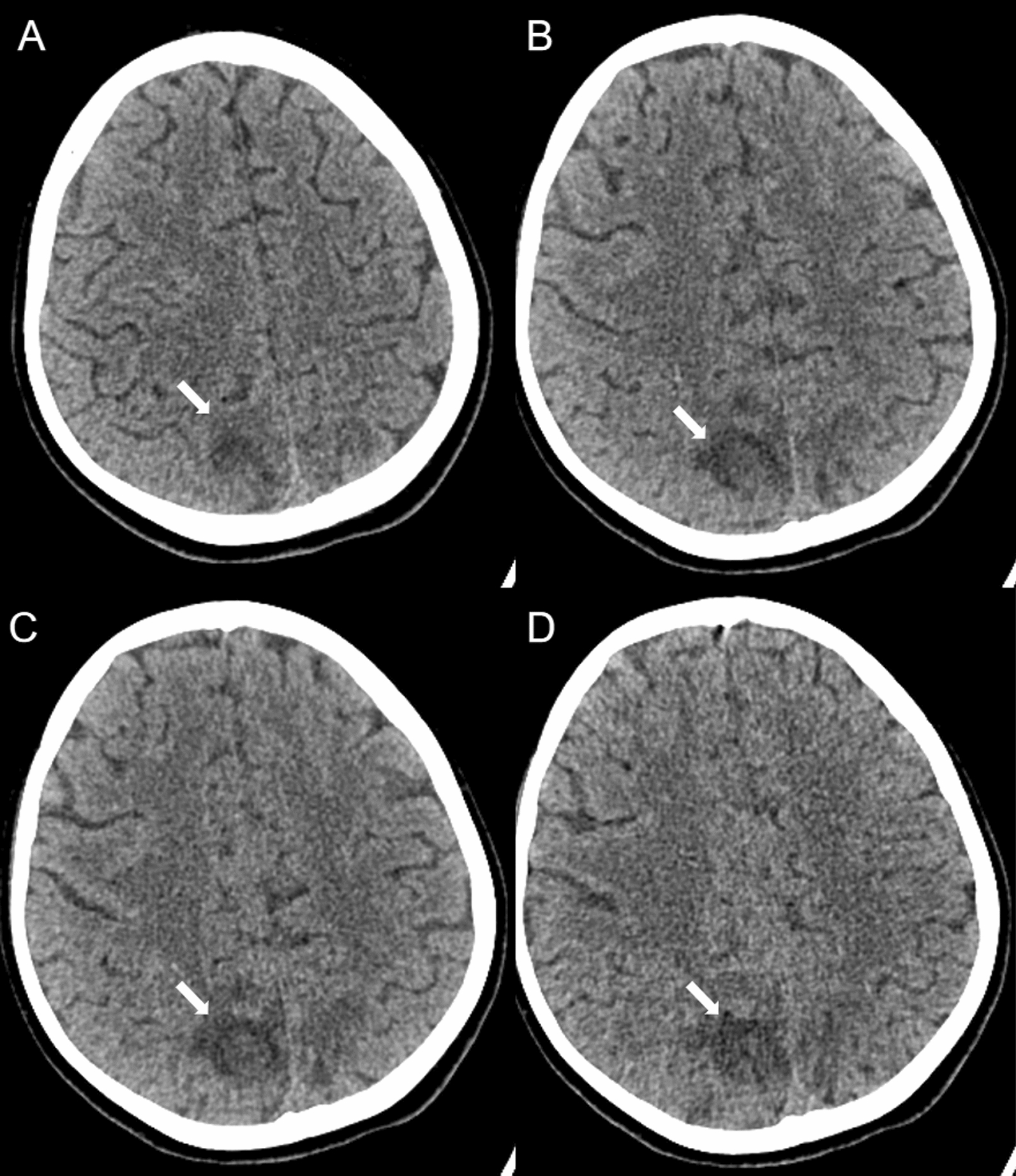
The CT manifestation of PRES, **A-D **:Bilateral Pariedtal-Occipital lobe cortex and subcortical patchy low-density shadows(the white arrow)

**Fig. 2:**
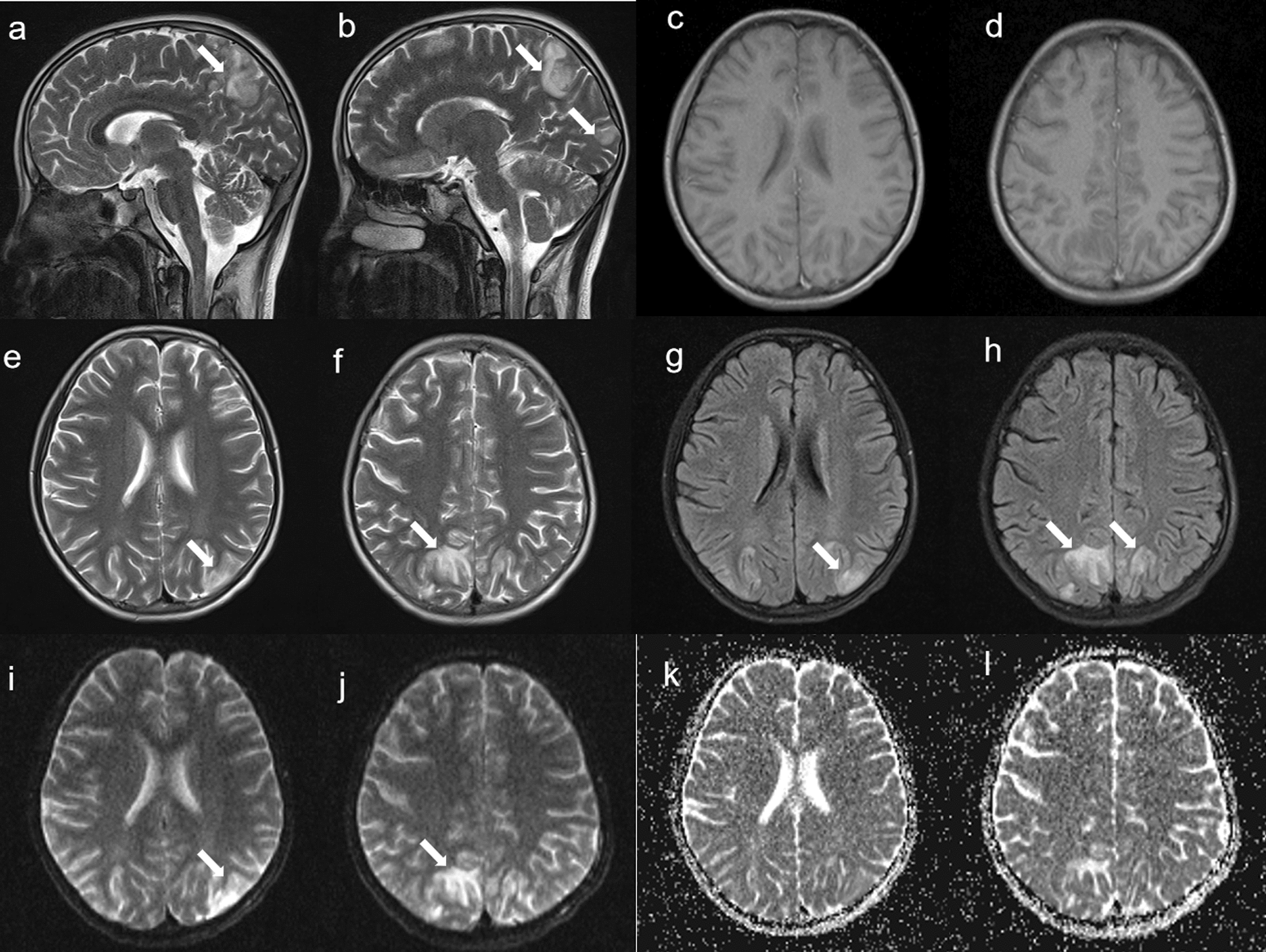
The MRI manifestation of PRES, **a-b** and **e–f**: Sagittal and Axial T2WI show predominantly subcortical abnormal (the white arrow); **g** and **h**:Axial T2 fluid-attenuated inversion recovery sequences show predominantly subcortical abnormal T2 signal (the white arrow); **i-l**: Diffusion-weighted image showing elevated regional apparent diffusion coefficient in a pattern typical of posterior reversible encephalopathy syndrome

The patient was a 10-year-old girl with a 5-month history of illness who initially presented with facial, palmar and plantar rashes, along with bilateral hand joint pain, followed by generalized edema. Upon admission, the patient was 152 cm in height, weighed 50 kg, and had a blood pressure of 140/82 mmHg (measured without prior pharmacological intervention). On the second day, following albumin administration and furosemide injection, the blood pressure was 128/82 mmHg.Physical examination revealed vasculitis-like rashes on both hands and feet, ischaemic necrosis of the toes, facial erythema, and bilateral lower extremity edema. The laboratory findings revealed a white blood cell count of 8.5 × 10⁹/L, a haemoglobin level of 120 g/L, a platelet count of 155 × 10⁹/L, normal liver and kidney function, and a serum albumin level of 20.5 g/L. The 24-h urine protein concentration was 11.36 g/L, and urinalysis revealed positive dysmorphic red blood cells and haematuria. Immunological tests revealed the following results: ANA titre of 1:1000 (homogeneous pattern), Anti-SSA antibody: 47 RU/mL, Anti-SSB antibody: 139.4 RU/mL, anti-ribosomal P protein antibody: 398.01 RU/mL, Anti-PM-Scl antibody: 96.34 RU/mL, Anti-Scl-70 antibody: 29.28 RU/mL, Anti-dsDNA antibody: 1:320, complement 3(C3): 0.12 g/L, complement 4(C4): 0.04 g/L, immunoglobulin G(IgG): 7 g/L, immunoglobulin M(IgM): 1.38g/L, immunoglobulin A(IgA): 2.43 g/L, anticardiolipin antibodies(ACA) and anti-neutrophil cytoplasmic antibody(ANCA) were negative, and an erythrocyte sedimentation rate of 46 mm/h. The patient’s SLE Disease Activity Index (SLEDAI) score was 26, which was based on findings of vasculitis, arthritis, haematuria, proteinuria, rash, low complement levels, and positive anti-dsDNA antibodies. Brain MRI at that time was normal. The patient was treated with intravenous methylprednisolone sodium succinate at 120 mg daily, cyclophosphamide 0.2 g every other day for seven doses, enoxaparin sodium injection (0.2 ml subcutaneously daily), and hydroxychloroquine sulfate for 15 days. Following this regimen, the rashes lessened, joint pain resolved, and oedema improved. However, the patient subsequently developed headaches, blurred vision, and seizures, with a blood pressure of 145/96 mmHg. Brain Computed tomography (CT) (Fig. 1) revealed irregular patchy hypodense areas in the bilateral parietal and occipital lobes. That evening, the patient experienced another episode of blurred vision in the right eye and partial vision loss in the left eye, which lasted for approximately 4 min before recovering and was accompanied by macroscopic haematuria and fever. Her blood pressure was 150/103 mmHg. Follow-up brain MRI revealed swelling and diffuse restriction of the gyri in the bilateral occipital and parietal lobes, which was consistent with systemic lupus erythematosus-associated posterior reversible encephalopathy syndrome (Fig. 2). Rituximab was suggested as a treatment, but parental consent could not be obtained.The patient was treated with antihypertensives, mannitol injection, fructose and sodium chloride injection to reduce intracranial pressure, and a regimen of 40 mg daily methylprednisolone sodium succinate, weekly subcutaneous telitacicept 160 mg, cyclophosphamide 0.6 g every two weeks, and hydroxychloroquine sulfate was used. The patient's headache and blurred vision disappeared, and her blood pressure gradually returned to normal. Follow-up brain MRI was normal after 6 months, and her SLEDAI score had decreased to 8.

## Discussion

PRES is a clinicopathological syndrome characterized by acute brain endothelial injury, disruption of the blood‒brain barrier, and vasogenic oedema. It was first described in 1996 and has since gained recognition [2]. The exact pathophysiological mechanisms remain unclear, but several hypotheses have been proposed, including "vasogenic," "cytotoxic," "immunogenic," and "neuropeptide/vasoconstrictor"theories. The vasogenic theory suggests that severe hypertension, exceeding the brain's autoregulatory mean arterial pressure (MAP) limit (typically > 140–150 mmHg), leads to blood‒brain barrier disruption and subsequent vasogenic oedema. The cytotoxic theory posits that various medications, such as chemotherapeutic agents (e.g., cyclophosphamide, vincristine) or immunosuppressants (e.g., tacrolimus, cyclosporine), can increase the levels of neurotoxins, triggering endothelial dysfunction. The immunogenic theory highlights the role of the immune system in the brain, resulting in endothelial injury, increased permeability, and vasogenic oedema mediated mainly by activated T cells and the release of cytokines (e.g., histamines, free radicals, and nitric oxide). Finally, the neuropeptide theory indicates that the release of potent vasoconstrictors, such as endothelin-1 and thromboxane A2, can lead to severe cerebral vasoconstriction, followed by endothelial dysfunction and hypoperfusion. If not reversed, this may result in brain oedema and ischaemia. Ultimately, all these theories converge to cause endothelial dysfunction, subsequent blood‒brain barrier breakdown, local hypoperfusion, and vasogenic oedema, leading to a range of clinical symptoms and imaging findings [3, 4].

PRES can occur in patients with autoimmune diseases, but there are few reports of its occurrence in systemic lupus erythematosus (SLE) patients compared with those of other rheumatic diseases. In children, the occurrence of PRES in SLE patients is even rarer. Romergryko et al. reported that the incidence of PRES in SLE patients is approximately 0.7%, with an estimated incidence of 0.04% in the paediatric population [5]. We describe a case of systemic lupus erythematosus in a child with lupus nephritis, cutaneous vasculitis, arthritis, high disease activity, and secondary hypertension. The patient received treatment with corticosteroids and cyclophosphamide, during which she developed PRES. Initially, high disease activity and the use of corticosteroids and cyclophosphamide were suspected as potential causes for PRES. However, after continuing corticosteroids and cyclophosphamide, along with the addition of telitacicept, the patient's condition improved, and a second MRI examination of the head after 6 months revealed normal results. These findings suggest that high disease activity likely contributes to the development of PRES.

In our review of 16 paediatric SLE patients with PRES (Tables 1 and 2), the male-to-female ratio was 3:13. All the children had seizures, and some had symptoms such as headaches, changes in consciousness, vision changes, and hemiplegia. Head MRI mainly involves the occipital and parietal lobes, followed by the temporal and frontal lobes and cerebellum, and the brainstem and basal ganglia less frequently. Two children experienced recurrence after active treatment, two patients were not followed up, Except for one case with persistent cognitive disturbance and the other remaining patients recovered without any neurological sequelae. LN, high disease activity, and hypertension are the main factors that induce PRES, followed by the use of glucocorticoids and immunosuppressants, and very few cases are caused by haemodialysis, plasma exchange, and rituximab. Augustine Manadan’s study indicated that SLE patients with nephritis are more likely to develop PRES than those without nephritis [6]. SM Jung's research further suggested that renal insufficiency and lupus nephritis significantly increase the likelihood of developing PRES [7]. A study from Chiang Mai University, Thailand, highlighted that SLE patients with lupus nephritis, anaemia, and hypertension are at greater risk for developing PRES, although most patients have a favourable recovery prognosis [8].

**Table 1:** Clinical characteristics of 16 children with PRES in juvenile systemic lupus erythematosus

Author	Gender/age (year)	Duration (month)	Diagnosis	BP (mmHg)	Clinical features	Vasogenic oedema sites	Triggers	SLEDAI	Neurological outcome	Outcomes
SerhatEmeksiz [11]	M/13	NA	SLE,LN	160/95	AC,SEZ,HCE,V	PL,TL	Cor	NA	Recovery	No further PRES clinically inactive
ChenTH [12]	F/14	NA	SL,LN	210/132	MC, SEZ, HCE,Pe	PL,FL,BG, TL,Cc	HTN,HD	NA	Recovery	No further PRES clinically inactive
	F/17	NA	SLE,LN	210/125	MC, SEZ, HCE	PL,TL,OL, FL(F),Cbl,BS,Bg	HTN,CTX,Cor	NA	Recovery	No further PRES clinically inactive
	F/16	NA	SLE,ATM	175/112	MC, SEZ, HCE,V	PL,OL,FL(F),Cc	HTN,Cor, PE	NA	Recovery	No further PRES clinically inactive
Baisya B [13]	F/7	7	SLE,LN,	170/90	HCE, SEZ, HP(L)	Cbl(R),T-PL,F-PL	LN ,SLEDA	27	Recovery	Refractory nephritis with repeat episode of PRES
	F/11	0.5	SLE,LN	130/80	SEZ, AC	OL(R), TL(B), PL(B),FL	SLEDA	20	Recovery	No further PRES clinically inactive
	F/13	12	SLE,LN	170/130	SEZ, coma	OL,FL,PL	SLEDA	21	Recovery	No further PRES clinically inactive
	M/15	36	SLE,LN, NPSLE(ADEM)	160/140	HCE, VOM、SEZ, hypoesthesia	TL(P),OL	LN, SLEDA	15	persistent cognitive disturbance	Repeat episode of PRES
	M/15	12	SLE,LN	150/100	SEZ	OL	LN, SLEDA	14	Recovery	Not on follow up
	F/16	60	SLE,LN	160/100	SEZ	OL	LN, SLEDA	20	Recovery	Not on follow up
Budhoo A [14]	F/13	1	SLE,LN,RF	HTN	SEZ,V,HCE	OL,P-FL	HTN, Elevated Cr,LN, MMF	21	Recovery	No further PRES clinically inactive
Punarol M [15]	F/10	6	SLE, anemia	160/？	V,gaze,HP,SEZ,AC	P-OL	DA,IVMP	15	Recovery	No further PRES clinically inactive
	F/13	84	SLE,LN, TTP, anemia, ARF	145/85	SEZ, AC	Cbl,Bs	RTX,LN,anemia,TTP	NA	Recovery	No further PRES clinically inactive
Ozyurek H [16]	F/13	NA	SLE	215/100	HCE,SEZ,AC	OL,PL(P)	CsA	NA	Recovery	No further PRES clinically inactive
Jung SM [7]	F/17	0.5	SLE,LN	200/100	HCE, SEZ	OL(B),TL	NA	45	Recovery	No further PRES clinically inactive
This case	F/10	5	SLE,LN, vasculitis	150/103	V,HCE,SEZ	P-OL,OL,PL	HTN,DA	26	Recovery	No further PRES clinically inactive

*F* Female, *M* male, *BP* blood pressure, *SLE* Systemic lupus erythematosus, *LN* Lupus nephritis, *ARF* acute renal failure, *TTP* thrombotic thrombocytopenic purpura, *ATM* acute transverse myelitis, *NPSLE* Neuropsychiatric lupus, *Pe* pulmonary edem, *FL* Frontal lobe, *PL* Pariedtal lobe, *OL* Occipital lobe, *P-OL* Pariedtal-Occipital lobe, *TL* Temporal lobe, *Cbl* Cerebellum, *Thal* thalamus,Bs:brain stem, *BG* Basal ganglia, *Cc* Corpus callosum, *P-FL* Pariedtal-Frontal lobe, *V* visual disturbance, *H* headaches, *MC* mental changes, *B* bilateral, *R* right, *L* left, *F* front, *P* posterior, *HTN* hypertension, *HCE* headache, *VOM* vomiting, *SEZ* seizure, *AC* Altered consciousness, *HP* hemiplegia, *Cor* Corticosteroids, *MMF* mycophenolate mofetil, *CsA* Cyclosporine, *IVMP* intravenous methylprednisolone, *PE* plasma exchange, *HD* hemodialysis, *ISDs* immunosuppressive drugs, *RTX* rituximab, *Cr* serum creatinine, *CR* complete resolution, *SLEDA* Systemic lupus erythematosus disease activity, *NA* not available

**Table 2:** Head MRI involvement sites and triggering factors in 16 Juvenile SLE patients with PRES

Involvement sites	n%	Triggering factors	n%
Occipital lobe	12 (75.0)	Lupus nephritis	8 (50.0)
Pariedtal lobe	11 (68.8)	High SLEDAI Score	6 (37.5)
Temporal lobe	6 (37.5)	hypertension	5 (31.3)
Frontal lobe	7 (43.8)	Corticosteroids	4 (25.0)
Cerebellum	3 (18.8)	ISDs	3 (18.8)
Brain stem	2 (12.5)	RTX	1 (6.3)
Basal ganglia	1 (6.3)	Others	3 (18.8)

*ISDs* immunosuppressive drugs, *RTX* rituximab, Others include plasma exchange and hemodialysis, *SLEDAI* Systemic Lupus Erythematosus Disease Activity Index

In a review, the prevalence of PRES in patients with systemic lupus erythematosus (SLE) ranged from 0.7% to 1.4%, with a recurrence rate of up to 13%. Risk factors include SLE activity, hypertension, haematologic conditions, and renal diseases, with renal disease being the most common; approximately 80% of patients experience hypertension secondary to LN. Typically, symptoms of PRES resolve within 24 h. In SLE patients, particularly those with high disease activity, significant endothelial dysfunction may occur, which is likely linked to elevated SLEDAI scores and increased levels of proinflammatory cytokines such as TNF-α, IL-1, IL-6, and interferon (IFN)-α. Interestingly, PRES can occur even without hypertension; the inflammatory and cytotoxic environment in SLE patients can explain its occurrence in the absence of severe hypertension. Fluctuations in blood pressure, often due to renal damage, can trigger PRES [9].

In children, the incidence of PRES is lower than that in adults, but hypertension is common. Owing to lower thresholds for cerebral blood flow autoregulation in children, average blood pressure at the onset of PRES symptoms is often lower than that in adults. In adults, the lower limit for cerebral blood flow autoregulation is approximately 50–60 mmHg, whereas in children, it averages approximately 40 mmHg. Seizures are frequent in paediatric cases of PRES [10], and in our reviewed cases, all presented with seizures, along with other symptoms such as headaches, visual disturbances, and focal neurological deficits. Radiological findings in children are similar to those in adults, typically involving the parietal and occipital lobes, but can also affect other areas, such as the temporal lobe, frontal lobe, cerebellum, and basal ganglia.

Most cases of PRES can achieve complete recovery with prompt treatment. Early detection and intervention are crucial, as delayed treatment can lead to irreversible neurological damage, such as cerebral haemorrhage or infarction. A small percentage of PRES patients may present with coma, elevated intracranial pressure, widespread brain oedema, and herniation syndromes, which pose life-threatening risks [17]. If the high disease activity of systemic lupus erythematosus leads to PRES, active treatment of SLE is necessary. If hypertension occurs, active antihypertensive treatment is needed [18].

In summary, PRES is less common in children with SLE, with primary triggers likely including high disease activity, lupus nephritis, renal hypertension, and renal insufficiency. In a minority of cases, medications such as corticosteroids, immunosuppressants, rituximab, or plasmapheresis may contribute. Clinical manifestations primarily include seizures, headaches, visual disturbances, vomiting, altered consciousness, and hemiparesis. Brain MRI is the most valuable diagnostic tool, often showing involvement of the parietal and occipital lobes, but other regions can also be affected. Early detection and aggressive treatment generally lead to favourable outcomes, with most patients fully recovering without neurological sequelae; however, delays in diagnosis can result in complications such as haemorrhage, infarction, and even mortality.

## Data Availability

All data can be download on the journal as of the date of publication. The data presented in this study are available on request from the corresponding author due to data privacy protection.
